# Clearance of senescent cells during cardiac ischemia–reperfusion injury improves recovery

**DOI:** 10.1111/acel.13249

**Published:** 2020-09-29

**Authors:** Emily Dookun, Anna Walaszczyk, Rachael Redgrave, Pawel Palmowski, Simon Tual‐Chalot, Averina Suwana, James Chapman, Eduard Jirkovsky, Leticia Donastorg Sosa, Eleanor Gill, Oliver E Yausep, Yohan Santin, Jeanne Mialet‐Perez, W Andrew Owens, David Grieve, Ioakim Spyridopoulos, Michael Taggart, Helen M. Arthur, João F. Passos, Gavin D. Richardson

**Affiliations:** ^1^ Biosciences Institute Newcastle University Newcastle upon Tyne UK; ^2^ School of Environmental Sciences Faculty of Science Agriculture & Engineering Newcastle University Newcastle upon Tyne UK; ^3^ Faculty of Pharmacy Charles University Prague Czech Republic; ^4^ School of Medicine Dentistry and Biomedical Sciences Centre for Experimental Medicine Institute for Health Sciences Queen`s University Belfast Belfast UK; ^5^ INSERM I2MC University of Toulouse Toulouse France; ^6^ Translational and Clinical Research Newcastle University Newcastle upon Tyne UK; ^7^ Department of Physiology and Biomedical Engineering Mayo Clinic Rochester MN USA

**Keywords:** cardiac, ischemia–reperfusion, remodeling, senescence, senolytic

## Abstract

A key component of cardiac ischemia–reperfusion injury (IRI) is the increased generation of reactive oxygen species, leading to enhanced inflammation and tissue dysfunction in patients following intervention for myocardial infarction. In this study, we hypothesized that oxidative stress, due to ischemia–reperfusion, induces senescence which contributes to the pathophysiology of cardiac IRI. We demonstrate that IRI induces cellular senescence in both cardiomyocytes and interstitial cell populations and treatment with the senolytic drug navitoclax after ischemia–reperfusion improves left ventricular function, increases myocardial vascularization, and decreases scar size. SWATH‐MS‐based proteomics revealed that biological processes associated with fibrosis and inflammation that were increased following ischemia–reperfusion were attenuated upon senescent cell clearance. Furthermore, navitoclax treatment reduced the expression of pro‐inflammatory, profibrotic, and anti‐angiogenic cytokines, including interferon gamma‐induced protein‐10, TGF‐β3, interleukin‐11, interleukin‐16, and fractalkine. Our study provides proof‐of‐concept evidence that cellular senescence contributes to impaired heart function and adverse remodeling following cardiac ischemia–reperfusion. We also establish that post‐IRI the SASP plays a considerable role in the inflammatory response. Subsequently, senolytic treatment, at a clinically feasible time‐point, attenuates multiple components of this response and improves clinically important parameters. Thus, cellular senescence represents a potential novel therapeutic avenue to improve patient outcomes following cardiac ischemia–reperfusion.

## Graphical abstract

Myocardial infarction and subsequent ischemia–reperfusion injury initiate senescence in multiple cell populations in the peri‐infarct region of the myocardium. Production of the SASP drives myocardial inflammation which promotes myocardial remodeling and inhibits angiogenesis. Treatment with the senolytic navitoclax reduced myocardial senescence and the associated SASP, resulting in a reduced scar size and increased vascularization which ultimately improved cardiac function.

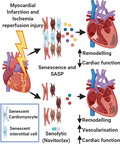

## 1 INTRODUCTION

Coronary heart disease (CHD) is the leading cause of death and disability in developed countries (Roger, 2007). The most serious manifestation of CHD is ST‐segment elevation myocardial infarction (STEMI), which is caused by acute blockage of a coronary artery leading to myocardial ischemia and cardiac cell death. The most effective intervention is timely reperfusion of the myocardium via primary percutaneous coronary intervention (Lonborg, 2015). Although early intervention can limit acute myocardial infarction (MI) injury, reperfusion can itself induce ischemia–reperfusion injury (IRI) resulting in adverse myocardial remodeling and an increased risk of progression to heart failure (Mozaffarian et al., 2016; Velagaleti et al., 2008). This necessitates the need to pursue the use of additional therapies to either prevent or ameliorate myocardial IRI, which has been described as a neglected therapeutic target (Hausenloy & Yellon, 2013).

Cellular senescence is defined as an irreversible cell‐cycle arrest characterized by dramatic alterations in gene and protein expression and the production of the senescence‐associated secretory phenotype (SASP) (Coppe et al., 2008). The SASP consists of a cocktail of pro‐inflammatory cytokines, chemokines, matrix proteases, and growth factors that if unhindered can induce many of the biological processes associated with maladaptive cardiac remodeling. These include attenuation of regeneration, induction of fibrosis and cellular hypertrophy and inflammation (Coppe et al., 2010). Furthermore, the SASP can induce senescence in surrounding healthy cells leading to the spreading of senescence throughout affected tissues (Acosta et al., 2013). Senescence can be induced by a variety of stresses including oxidative stress (Anderson et al., 2018; de Magalhaes & Passos, 2018).

A key component of IRI is the increased generation of reactive oxygen species (Chouchani et al., 2014) which is thought to contribute to tissue dysfunction. Previously, we have shown that cardiomyocyte (CM) senescence can be induced by oxidative stress, accumulates during aging (Anderson et al., 2019; Walaszczyk et al., 2019), and contributes to age‐related myocardial remodeling (Anderson et al., 2019). Furthermore, using the senolytic navitoclax (ABT263), a Bcl‐2 family inhibitor, we demonstrated that elimination of age‐related senescence prior to MI improved outcome in aged mice (Walaszczyk et al., 2019). These data indicate that pre‐existing senescence impairs recovery in aged animals; however, the possibility that senescence is induced during IRI and contributes to the disease pathophysiology has not been investigated.

In this study, we hypothesized that cellular senescence is an outcome of the oxidative burst occurring during cardiac IRI and is a key contributor to its associated adverse ventricular remodeling and impaired cardiac function. We show that cardiac ischemia–reperfusion (IR) induces myocardial senescence and expression of a pro‐inflammatory, profibrotic, and anti‐angiogenic SASP in young adult mice. Post‐IR, treatment with navitoclax clears senescent cells in the heart, attenuates SASP‐mediated inflammation, increases myocardial vascularization, reduces scar size, and leads to improved cardiac function. Our work provides proof‐of‐concept that targeting senescent cells may represent a new therapeutic avenue following cardiac IR.

## 2 RESULTS

### 2.1 Myocardial infarction with reperfusion induces senescence in multiple cardiac lineages

**Figure 1:**
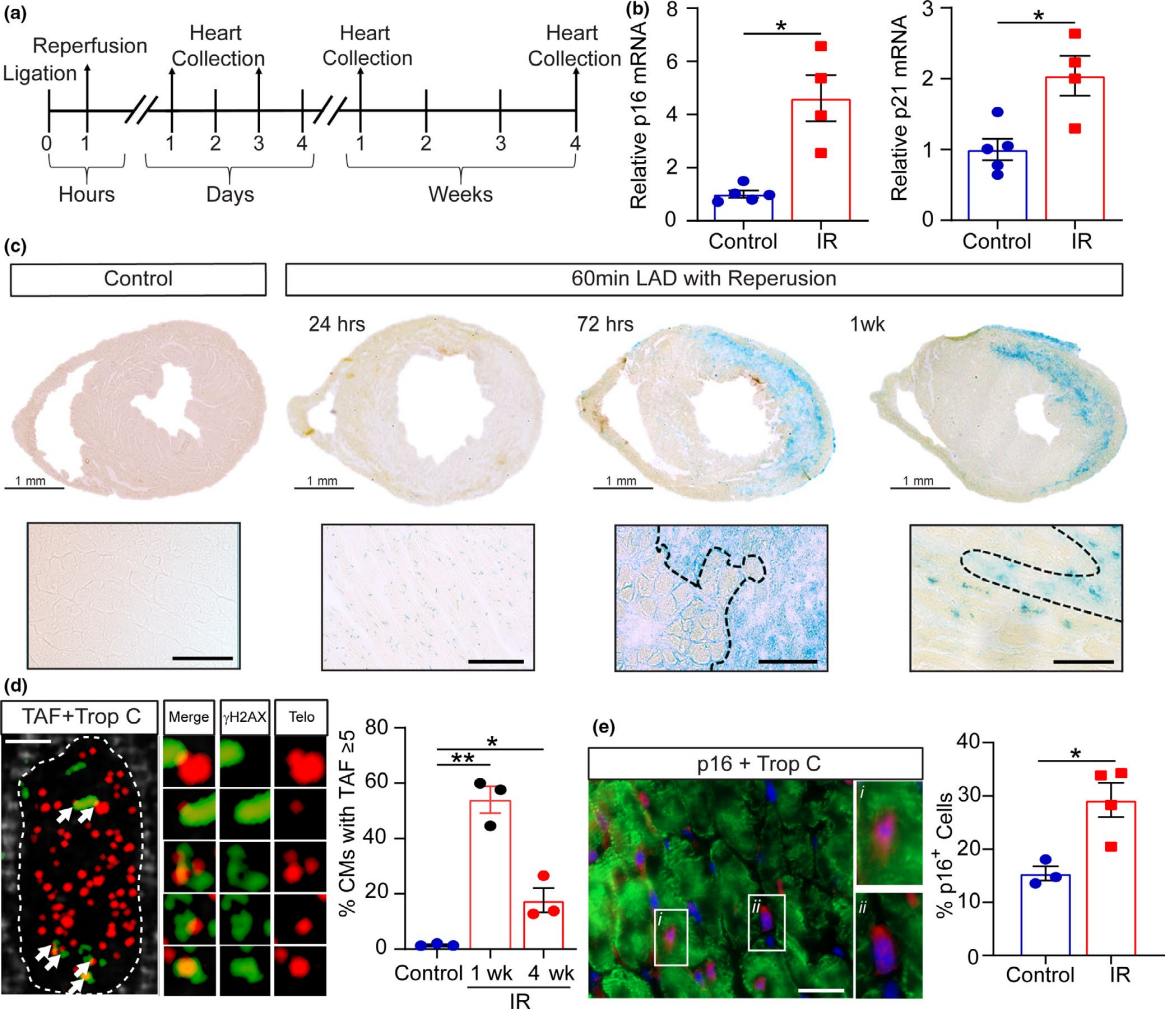
Cardiac ischemia–reperfusion induces cellular senescence. (a) Experimental design. Mice were either subjected to 60 minutes LAD‐ligation followed by reperfusion (IR) or received no injury (Control) and hearts collected at the indicated times. (b) At 4 weeks post‐IR, hearts were collected and the LV region apical to suture isolated. Real‐time qPCR gene expression analysis performed to determine the relative expression of p16 and p21 mRNA (normalized to GADPH). N ≥ 4/group. (c) SA‐β‐Gal staining in control and at 24 hours, 72 hours, and 1 week post‐IR. Lower panels) Higher magnification images of the peri‐infarct region. Scale bars 50 µm. (d) Representative images of TAF, γH2AX co‐localized with Telomere immuno‐FISH, in a trop‐C^+^ CM (trop‐C white, telo‐FISH red, γH2AX green). White arrows indicate individual TAF. Images are obtained from the z‐stacks of 10 μm sections. Right image panel) Higher magnification of 5 regions showing 6 individual TAF. The merged image demonstrates the co‐localization of γH2AX (green) and telomere (red). Individual red and green channels are also shown. Scale bar 2.5 μm. Right) The mean number of CMs with ≥5‐TAF at 1 and 4 weeks post‐IR. N = 3/group. (e) Left) Representative image of p16 expression at 4 weeks post‐IRI (p16 red, trop‐C green, DAPI blue. (*i*) shows a magnified example of a p16^+^ CM (trop‐C^+^), and (*ii*) is an example of a p16^+^ interstitial cell. Scale bar 20 µm. Right) Quantification of the percentage p16 expressing cells at 4 weeks post‐IR N ≥ 3/group. Data are mean ± SEM. B and E were analyzed by 2‐tailed unpaired t‐test; D was analyzed by a 1‐way ANOVA followed by Tukey's post hoc test, **p < 0.01; *p < 0.05

To determine whether senescence is induced in a clinically relevant model of cardiac IRI, 3‐ to 4‐month‐old male mice were subjected to 60 minutes LAD‐ligation followed by reperfusion and hearts were collected at different time‐points after reperfusion (Figure 1A). The qRT‐PCR analysis in hearts collected at 4‐weeks post‐IR demonstrated that mRNAs encoding senescence‐associated markers p16 and p21 were increased in the myocardium of IR injured mice compared to controls (Figure 1B). Furthermore, histological analysis showed an increased expression of senescent markers both within the infarct and in the peri‐infarct region of the left ventricular myocardium (Figure 1C–E and Figure S1A). At 72 hours, cells within the infarct and the peri‐infarct zone showed increased senescence‐associated β‐galactosidase activity (SA‐β‐Gal) (Figure 1C). SA‐β‐Gal activity was also evident 1‐week post‐IR throughout the infarct in interstitial cells and CMs (Figure 1C and Figure S1B). IRI is associated with increased oxidative stress (Peoples et al., 2019), and in line with this, we saw an increase in 4‐HNE staining (a marker of lipid peroxidation) in the infarct zone at 72 hours following IR, with the area demonstrating the highest levels of oxidative stress also displaying the highest senescence burden (Figure S2). During aging, senescence in CMs is characterized by the presence of telomere‐associated DNA damage foci (TAF) which can be induced by oxidative stress (Anderson et al., 2019). To ascertain if telomere dysfunction occurred in CMs following IR, we quantified TAF in CMs within the peri‐infarct region. We found that the frequency of CMs positive for ≥5 TAF was significantly increased in IR injured mice at both 1 week (1.53%±0.45 vs 54.0%±8.3) and 4 weeks post‐IR (17.71%±7.67) (Figure 1D). Following an assessment of p16 antibody specificity (see Methods and Figure S3 for details), we found a significant increase in the frequency of cells expressing p16 protein 4 weeks following IR compared with controls (Figure 1E). This included CMs, identified via co‐expression of troponin C (trop‐C), and cells of the interstitial population.

### 2.2 Navitoclax eliminates senescent cells and improves cardiac function following cardiac ischemia–reperfusion injury

**Figure 2:**
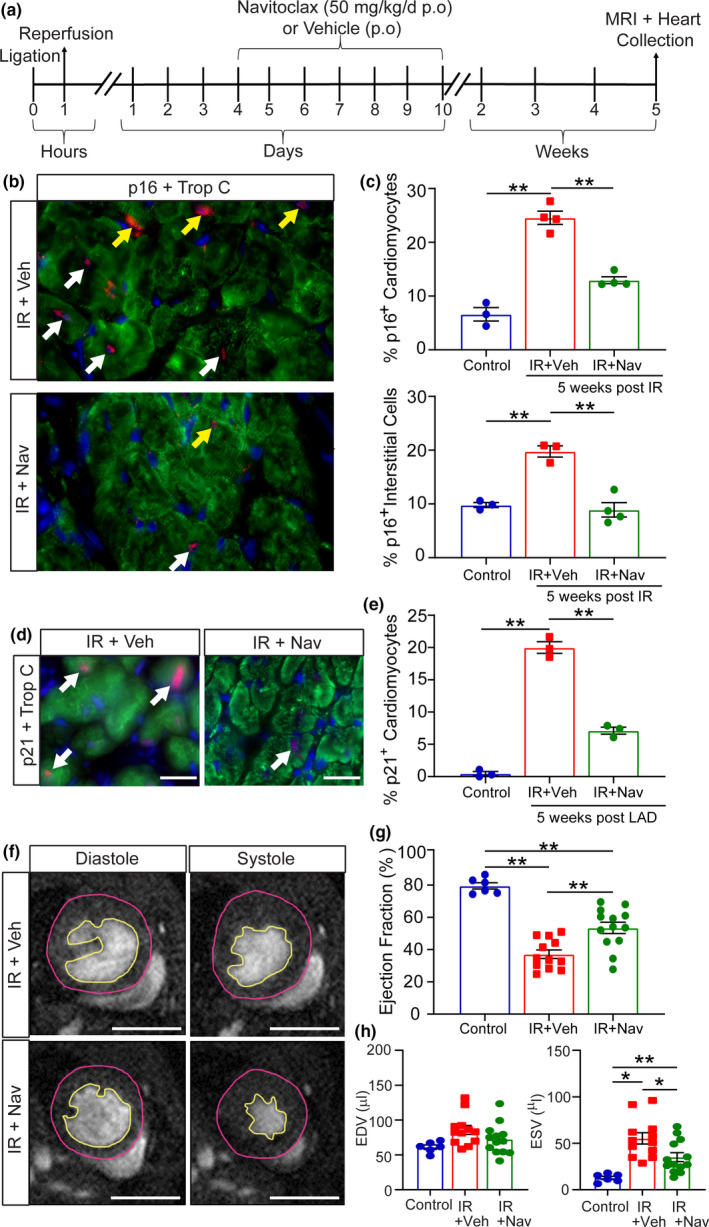
Pharmacological clearance of senescent cells following ischemia–reperfusion reduces cellular senescence and improves cardiac function. (a) Mice were either subjected to 60‐minute IR or received no injury (Control). After IR mice were randomly assigned to the vehicle (IR) or navitoclax (IR+Nav) groups and provided with navitoclax or vehicle daily from 4 days post‐IR for 7 days. MRI was performed at 5 weeks post‐IR, and hearts were collected. (b) Representative images of trop‐C^+^ CMs co‐expressing p16 (examples indicated by white arrows) and p16 expressing intestinal cells (examples indicated by yellow arrows) in the LV peri‐infarct region (p16 red, trop‐C green and DAPI blue). Scale bars 20 µm. (c) Quantification of the percentage trop‐C^+^ CMs and intestinal cells expressing p16^+^ in the LV peri‐infarct region. N ≥ 3/group. (d) Representative images of trop‐C^+^ CMs co‐expressing p21 in the LV peri‐infarct region (p21 red, trop‐C green, and DAPI blue). White arrows indicate examples of p16^+^ CMs. Scale bars 20 µm. (e) Quantification of the percentage of trop‐C^+^ CMs co‐expressing p21^+^ in the LV peri‐infarct region. N = 3/group. (f) Examples of individual short‐axis cine‐MR images of mouse hearts. Scale bars 5 mm. G and (h) EF%, EDV and ESV were calculated based on manual measurements of LV epicardial and endocardial borders. Measurements were made in all cine slices at end‐diastole and end‐systole. Graphs representing data obtained from MRI analysis. Control N = 6, IR N = 12 and IR+Nav N = 13. Data are mean±SEM. c, e, g, and H were analyzed by one‐way ANOVA followed by Tukey's post hoc test, **p < 0.01; *p < 0.05

To ascertain if the observed increase in senescence following cardiac IR contributes to detrimental pathophysiology and represents a valid therapeutic target to improve outcome, we investigated the effects of navitoclax treatment. At 4 days post‐IR, once senescence was established, mice were treated with either vehicle only or navitoclax for 7 consecutive days (Figure 2A). Immunohistochemical analysis of the LV at 1 month after navitoclax treatment showed a significant reduction in the proportion of p16^+^ CMs (24.56%±2.45 vs 6.60%±2.16) and p16^+^ interstitial cells (19.76%±1.82 vs 8.90%±2.66) in the peri‐infarct region of navitoclax‐treated compared to vehicle‐treated hearts (Figure 2B,C). A reduction in p21 expressing CMs in the peri‐infarct region (20.00% ±1.57 vs 7.12% ±0.94) was also observed at the same time‐point (Figure 2D,E). CMs with more than 5 TAF were also reduced in the peri‐infarct region in navitoclax‐treated animals (14.22% ±2.34 vs 6.41% ±1.63). Having demonstrated the effectiveness of navitoclax to reduce senescence, we next aimed to determine if there is a causal relationship between senescence and impaired cardiac function following IR. In a separate cohort of mice, cardiac MRI was performed at 5 weeks post‐IR in navitoclax and vehicle‐treated mice. All ligated mice demonstrated increased LV end‐systolic volumes (ESV) and a decrease in ejection fraction (EF) (Figure 2F–H). Mice treated with navitoclax showed a significantly higher EF than vehicle controls (Figure 2G) as a result of a better maintained LV systolic volume (Figure 2H). In addition, navitoclax‐treated mice displayed a significantly larger cardiac output and stroke volume compared to the vehicle group (Figure S5). No significant difference in EDV was observed between any experimental group (Figure 2H). These data support a role for senescence driven myocardial dysfunction following cardiac IR.

### 2.3 Proteome profiling indicates that modulation of molecular pathways involved in remodeling, inflammation, and respiration underlies navitoclax‐mediated improvement in cardiac function post‐IR

**Figure 3:**
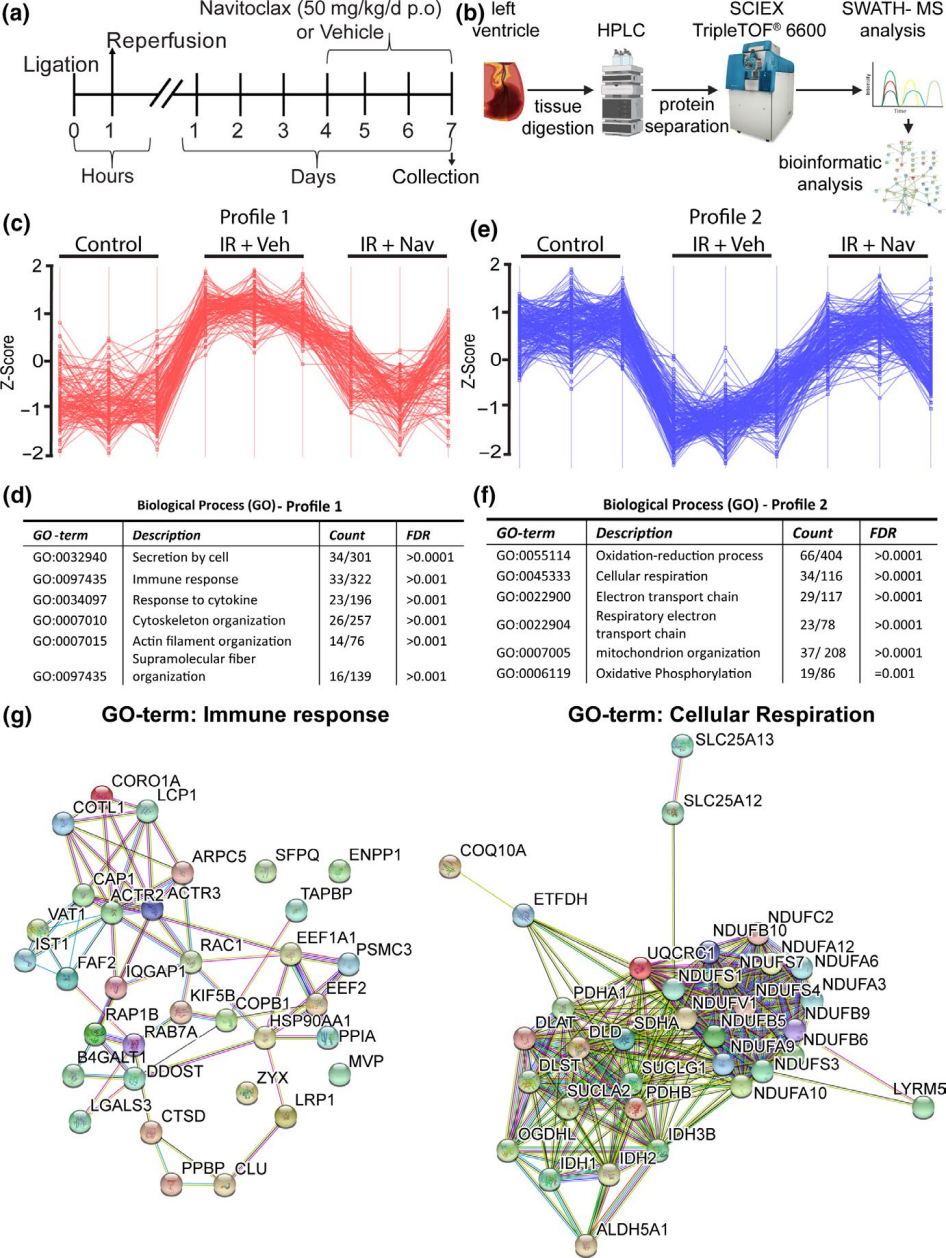
Proteomic pathway profiling demonstrates navitoclax modulates protein networks involved in remodeling, inflammation, and respiration. (a) Experimental design. Mice were either subjected to 60‐minute IR or received no injury (Control). After IR mice were randomly assigned to the vehicle (IR) or navitoclax (IR+Nav) groups and provided with navitoclax or vehicle daily from 4 days post‐IR for 4 days. On the fourth day of treatment, hearts were collected and the LV apical to the suture isolated for analysis (N = 3/group). For control mice, a comparable region of LV was isolated for the analysis. (N = 3). (b) Workflow for SWATH‐MS proteomics. (c) Perseus software was used to identify proteins that followed Profile 1. Vertical lines represent biological repeats in each of the indicated groups. Horizontal lines indicate individual proteins that followed the profile at an FDR < 0.01. (d) List of selected significantly enriched GO biological terms for the proteins identified to follow profile 1 at an FDR < 0.01. (e) Perseus software was used to identify proteins that followed Profile 2. Vertical lines represent biological repeats in each of the indicated groups. Horizontal lines indicate individual proteins that followed the profile at an FDR < 0.01. (f) List of selected significantly enriched GO biological terms for the proteins identified to follow profile 2 at an FDR < 0.01. (g) Examples of enriched protein networks for the GO term “immune response,” which followed profile 1 (increased following IR and attenuated by navitoclax treatment), and the GO term “cellular respiration” which followed profile 2 (decreased following IR and rescued by navitoclax treatment)

We next conducted proteomic profiling to identify the protein networks modified by IR as well as the protein networks that are modified by senescence clearance at 7 days post‐IR. This early time‐point was chosen to allow the identification of those networks that may contribute to the observed improvement in cardiac function rather than protein networks that are modified as a result of the observed improvement (Figure 3A). At this time‐point, a reduction in p21 expression was observed in the LV of navitoclax‐treated animals compared to vehicle controls, consistent with the elimination of senescence (Figure S6). Having demonstrated that navitoclax was reducing senescence by day 7 post‐IR, LC‐MS/MS analysis was performed on protein lysates obtained from LV tissue posterior to the suture (containing the infarct and surviving myocardium within this region of the LV) of vehicle control and navitoclax‐treated mice at 7 days post‐IR and comparable regions of LV from naïve, age‐matched control mice (Figure 3B). From a total of 3213 proteins quantified in these LV tissues, 162 were increased (T‐test, FDR<0.05) and 142 were reduced following IR. To identify the proteins/pathways modulated by navitoclax selected protein abundance profiles were analyzed to identify proteins increased by IR but attenuated following navitoclax treatment (profile1) (Figure 3C). 137 proteins were identified that followed this profile at an FDR <0.01 and 376 at an FDR of <0.05 (Data set 1). To assess the potential significance of these proteins to biological functions, pathway analysis was performed in string V 11.0, (Szklarczyk et al., 2019) for the 137 proteins matching the reference with FDR <0.01 (Figure S7). Enriched GO terms (biological processes) included processes related to inflammation, such as secretion by cell, cellular secretion, immune response, and response to cytokine (Figure 3D). Additionally, the analysis indicated that navitoclax treatment attenuated proteins involved in biological processes related to supramolecular fiber organization and cytoskeleton organization.

Next, we aimed to identify the proteins/pathways that followed profile 2 (decreased by IR and rescued following navitoclax treatment) **(**Figure 3E) and identified 199 proteins that followed this profile at an FDR <0.01 and 527 at an FDR of <0.05 (Data Set 2). Pathway analysis of the 199 proteins with an FDR <0.01 (Figure S8), identified GO terms enriched for this profile were related to cellular respiration and mitochondrial function including, oxidative phosphorylation and the electron transport chain (Figure 3F), suggesting that navitoclax treatment may improve in mitochondrial function and attenuate the oxidative stress caused by IR. Examples of protein networks for the GO term “immune response,” enriched in the proteins following profile 1 and “cellular respiration,” enriched in the proteins following profile 2, are shown in Figure 3G.

### 2.4 Navitoclax attenuates the SASP

**Figure 4:**
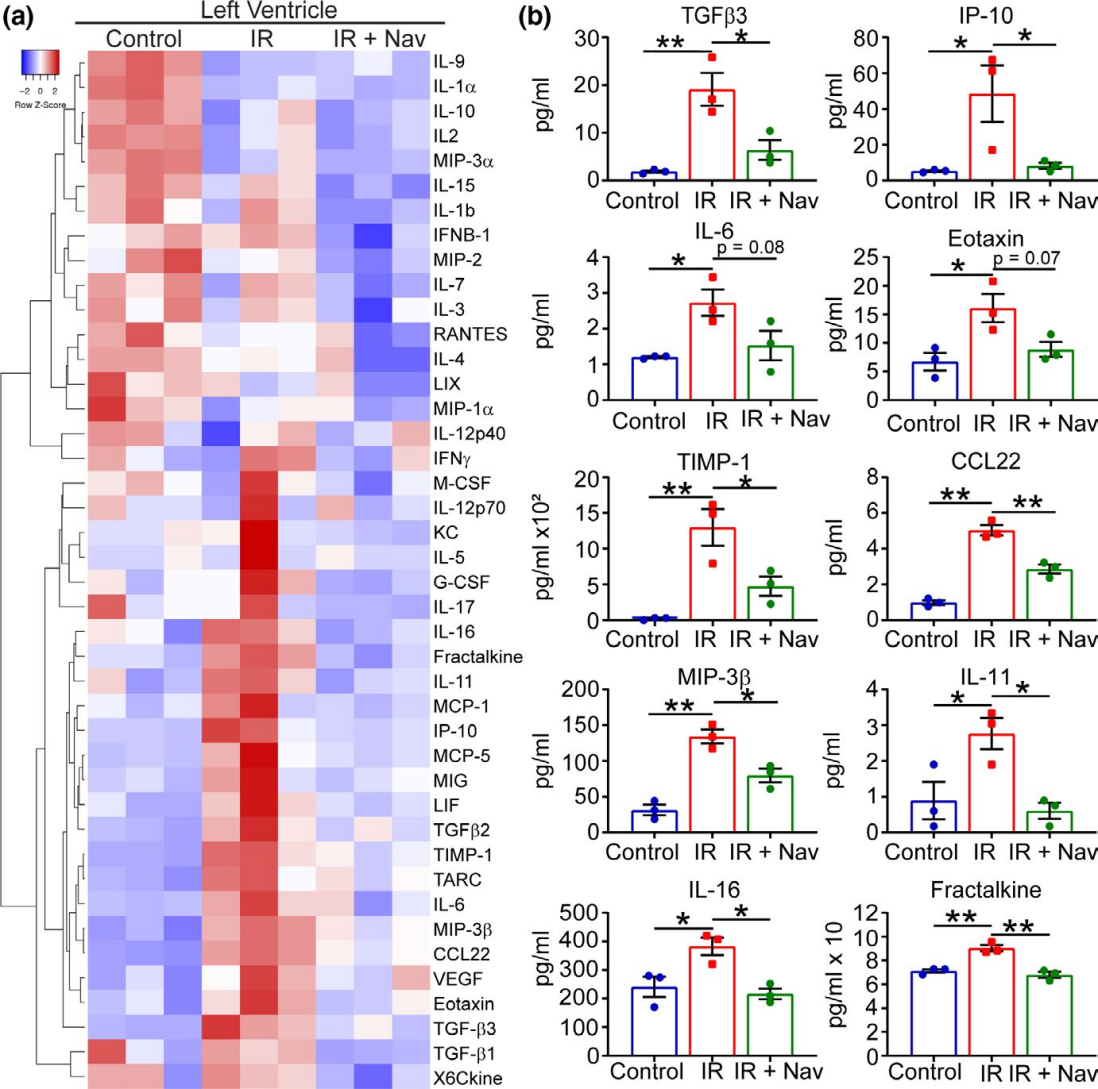
Navitoclax treatment attenuates the post‐ischemia–reperfusion inflammatory response. (a) Clustered heatmap showing all analyzed cytokine protein levels in the LV of naïve control (Control) and IR with either vehicle (IR) or navitoclax treatment (IR +Nav). (b) Expression of individual protein levels in the LV myocardium of heart in the indicated experimental groups. Data are mean ± SEM for each treatment group. N = 3/group. Analysis for all proteins was by one‐way ANOVA followed by Tukey's post hoc test, *p < 0.05, **p < 0.01

Pathway analysis of the proteomics data indicated a reduction of senescence resulted in the modulation of multiple protein networks associated with inflammation and cytokine activity. As such, it is attractive to hypothesize that elimination of senescent cells post‐IR improves outcome as a result of attenuation of a pro‐inflammatory and profibrotic SASP. To investigate this further a cytokine array was used to evaluate cytokines released within the LV of naïve control and IR hearts treated with either vehicle or navitoclax harvested at 7 days post‐injury, as in (Figure 3A). As previously, proteins were isolated from the LV tissue posterior to the suture (containing all the infarct and surviving myocardium) from vehicle and navitoclax‐treated IR and control mice to compare cytokine expression. The analysis revealed that IR caused an up‐regulation of SASP proteins including interleukin‐6 (IL‐6), interferon gamma‐induced protein 10 (IP‐10), eotaxin, and members of the TGF‐β superfamily. Importantly, these SASP proteins were reduced in the navitoclax‐treated IR animals (Figure 4A,B). In addition, interleukin‐11 (IL‐11), interleukin‐16 (IL‐16), CCL22, and MIP‐3β which have been previously associated with cardiac fibrosis, (Schafer et al., 2017; Tamaki et al., 2013) or cardiovascular disease (Kimura et al., 2018; Safa et al., 2016), and fractalkine (CX3CL1), a chemokine associated with poorer cardiac functional outcome and increased mortality in MI patients (Boag et al., 2015), showed a similar trend of reduction following navitoclax treatment. A complete list of cytokines and their expression levels is included in **Supplementary Table I**.

### 2.5 Navitoclax treatment reduces infarct size and promotes angiogenesis but not cardiomyocyte proliferation in vivo

**Figure 5:**
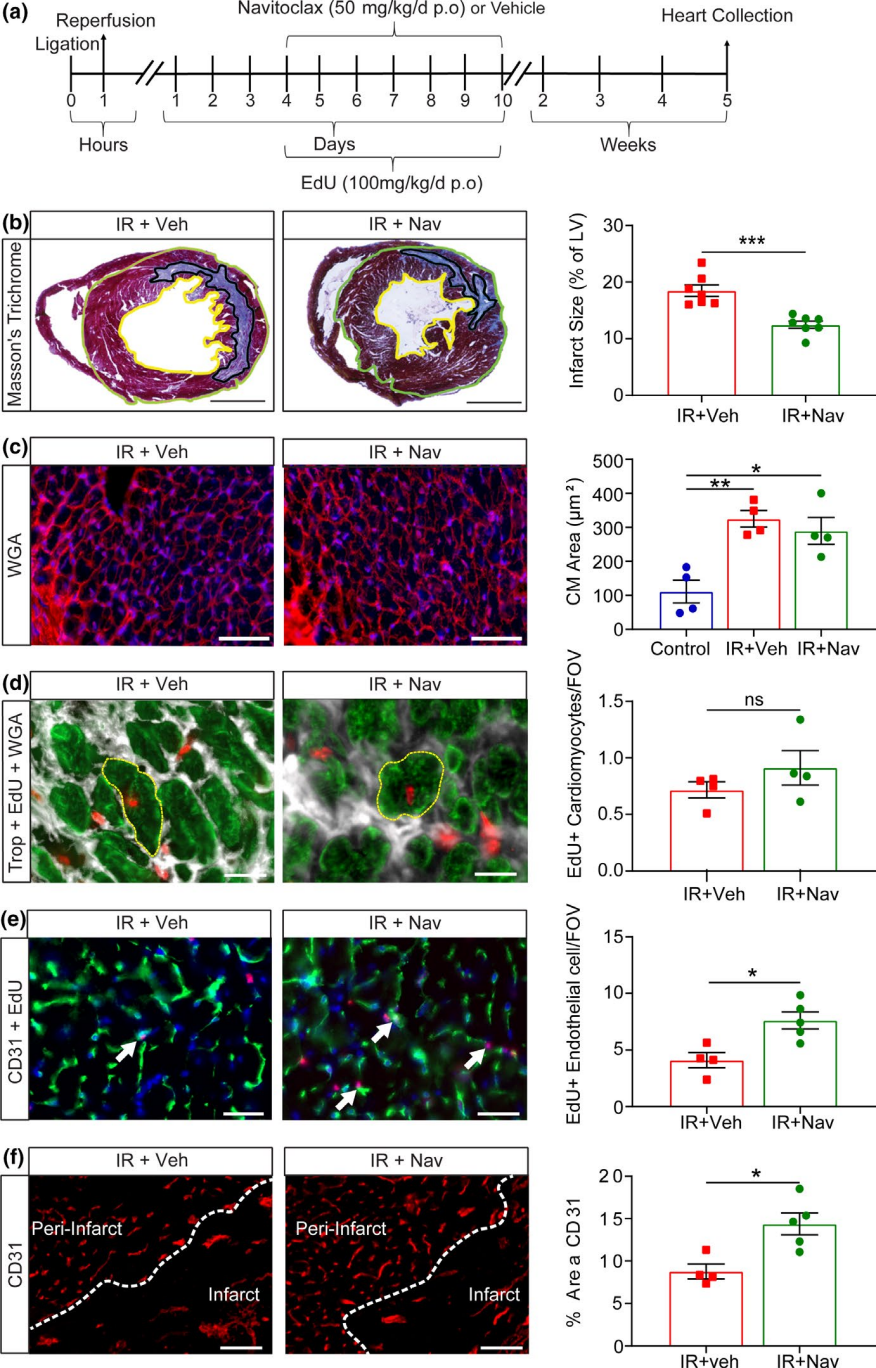
Navitoclax reduces scar size and increases angiogenesis but has no influence on hypertrophy or cardiomyocyte proliferation. (a) Experimental design. Mice were subjected to 60‐minute LAD‐Ligation with reperfusion and then at day 4 post‐IR were treated with vehicle (IR) or navitoclax (IR +Nav) daily for 7 days. Mice were also provided with EdU during the same period. Hearts were collected at 5 weeks post‐IR. (b) Representative image of Masson's trichrome staining. Scale bars 1 mm. Right) Quantification of infarct size relative to total LV area. N = 7/group. (c) Representative images of WGA staining for quantification CM cross‐sectional area. Scale bars 50 µm Right) CM cross‐sectional area μm^2^. N = 4/group, >150 CMs analyzed per mouse. (d) Representative image of trop‐C^+^ CMs co‐labeled with EdU highlighted in yellow (Trop‐C green, EdU Red and WGA white). Scale bars 20 µm Right) Quantification of trop‐C^+^/EdU^+^ cells per field of view in the peri‐infarct region. N = 4/group. (e) Representative image of CD31^+^ expressing endothelial cells co‐labeled with EdU (trop‐C green, EdU Red). White arrows indicate co‐labeled cells. Scale bars 20 µm. Right) Quantification of the total number of CD31^+/^EdU^+ ^cells per field of view in the peri‐infarct region. N ≥ 4/group. (f) Vessel density in the peri‐infarct and infarct zone analyzed using CD31 immunostaining. Scale bars 50 µm. Right) Quantification of the percentage area of CD31 expression. N ≥ 4/group. Data are mean ± SEM; b, d, e, and f were analyzed 2‐tailed unpaired t‐test; and C was analyzed by one‐way ANOVA followed by Tukey's post hoc test, ***p < 0.001; **p < 0.01; *p < 0.05

Navitoclax treatment post‐IR reduces expression of SASP proteins with established roles in myocardial remodeling and attenuates biological pathways related to inflammation, ECM production, and cytoskeletal organization. These findings led us to hypothesize that the elimination of senescence and its associated SASP leads to improved cardiac recovery via a reduction in adverse myocardial remodeling. Mice were treated as previously and also provided EdU to allow quantification of proliferation (Figure 5A). In line with our hypothesis, scar size measured by Masson's trichrome staining was significantly reduced in the navitoclax‐treated mice compared to vehicle control at 5 weeks post‐IR** (**12.47%±1.68 vs 18.50%±2.72 Figure 5B). IR also resulted in a significant increase in CM size; however, no difference was observed between the navitoclax‐treated and vehicle groups (Figure 5C). Accumulation of senescence and expression of the SASP could also impact on regeneration via the bystander effect. To investigate *de novo* CM regeneration following navitoclax treatment we quantified EdU positive cells in combination with the CM marker trop‐C and cell membrane marker wheat germ agglutinin (WGA) in the peri‐infarct region of the myocardium. We found a trend toward an increase in EdU labeled CMs following navitoclax; however, this was not significant (0.91 ± 0.30 cells per FOV vs 0.72 ± 0.14 cells per FOV, Figure 5D).

Proteomic data analysis also indicated that the elimination of senescent cells and the SASP is associated with improved respiration. This together with the documented anti‐angiogenic activities of SASP proteins, including IP‐10 (Campanella et al., 2010), led us to further hypothesize that subsequent to IR SASP inhibits endothelial proliferation leading to reduced angiogenesis. Accordingly, a navitoclax‐mediated reduction in senescence and SASP would attenuate this effect and allow for increased angiogenesis. Histological analysis of EdU together with the endothelial cell marker CD31 showed a significant increase in endothelial cell proliferation in the navitoclax‐treated animals compared with vehicle controls (7.62 ± 1.67 cells per FOV vs 4.11 ± 1.3 cells per FOV, Figure 5E). To establish if this could be a result of SASP driving endothelial cells to senescence, we performed *in vitro* conditioned media experiments. Conditioned medium isolated from senescent fibroblasts reduced cardiac endothelial cell proliferation (measured by Ki67 expression and cell output) and increased endothelial superoxide production, a characteristic of endothelial cell dysfunction (Figure S9). Furthermore, endothelial cells treated with conditioned media from senescent cells demonstrated increased p21 but not p16 expression (Figure S9F). Finally, we quantified peri‐infarct region vascularity. Consistent with an *in vivo* pro‐angiogenic effect (Redgrave et al., 2017), the peri‐infarct region of hearts from animals treated with navitoclax had significantly increased vessel density in the peri‐infarct zone compared vehicle control (14.38%±2.88% vs 8.87%±1.74, Figure 5F). Taken together, our data indicate that in young animals cardiac IR drives senescence and a pro‐inflammatory, anti‐angiogenic, and profibrotic SASP. Moreover, navitoclax mediates the reduction in senescence and improves cardiac function by attenuating SASP, reducing scar size, and enabling increased angiogenesis.

## 3 DISCUSSION

Efforts to target the oxidative insult that occurs immediately following IR using therapies such as antioxidants have proven unsuccessful (Baehr et al., 2019). The current study provides novel data demonstrating that navitoclax mediates elimination of the senescence that occurs downstream of oxidative stress and that this reduces scar size, increases angiogenesis, and improves cardiac function.

Subsequent to cardiac IR, the degree to which remodeling occurs is not only dependent on the immediate detrimental processes associated with ischemia and rapid oxidative stress due to reperfusion but is also dependent on the outcomes of a complex inflammatory response (Sánchez‐Hernández et al., 2020). Tissue damage caused by IR induces an acute inflammatory response responsible for the elimination of necrotic debris (Prabhu & Frangogiannis, 2016). In mice, this initial response begins to abate at around 5 days, being replaced by a reparative and proliferative phase which functions to suppress the acute response and coordinates tissue remodeling (Sánchez‐Hernández et al., 2020). While both phases of inflammation are required for successful wound healing they can also contribute to myocardial dysfunction. The acute inflammatory response can expand tissue damage and a severe or prolonged reparative response is associated with pathological scarring fibrosis (Prabhu & Frangogiannis, 2016).

Using a SWATH‐MS based proteomic approach we have demonstrated that navitoclax‐mediated reduction of senescence results in the attenuation of multiple biological processes associated with inflammation and immunity. Furthermore, cytokine array analysis identified that this attenuation included reduced expression of cytokines associated with the nuclear factor‐κB (NF‐κB) signaling pathway including CCL22, IL‐6, IL‐11, IP‐10, eotaxin, and fractalkine (Bhavsar et al., 2008; Bitko et al., 1997; Hein et al., 1997; Nakayama et al., 2004; Shultz et al., 2007; Son et al., 2008). These proteins are not only typical of the SASP (Salminen et al., 2012) but have also been identified as modulators of the acute inflammatory phase following IR (Boag et al., 2015). Mouse models in which the NF‐κB signaling pathway or individual NF‐Κb‐mediated proteins are reduced demonstrate decreased pathological remodeling, a reduction in scar size and improved vascularization (Campanella et al., 2010; Obana et al., 2010; Prabhu & Frangogiannis, 2016). Conversely, overexpression of NF‐κB associated cytokines, such as IL‐6, exhibit adverse remodeling and heightened myocardial inflammation (Hilfiker‐Kleiner et al., 2010). Our data suggest that senescence and the SASP contribute directly to acute inflammation post‐IR and senolytics, such as navitoclax, provide a means to attenuate the production of multiple cytokines and chemokines, known to be detrimental to recovery.

As senescence was evident by 3 days post‐IR, we treated mice from this time‐point using a dose of navitoclax that we have previously shown to induce myocardial senolysis (Anderson et al., 2019). This treatment extended into the reparative phase of the response to heart injury, a phase that is characterized by the secretion of TGF‐β1, an anti‐inflammatory and profibrotic cytokine (Hanna & Frangogiannis, 2019). TGF‐β expression is dynamic in the heart post‐IR, where it is critical for the switch from the pro‐inflammatory to the resolution phase of cardiac healing, and for driving formation of the fibrotic scar. In mice, TGF‐β1 and β2 expression peaks at 6–72 hours post‐reperfusion and declines after 3 to 7 days (Deten et al., 2001; Dewald et al., 2004). TGF‐β3 expression is induced at a later 3‐7 day time‐point and is maintained at high levels over a longer time‐frame. Early neutralization of TGF‐β signaling at 24 h post‐MI is detrimental as it increases both cardiac dysfunction and mortality, whereas late disruption of TGF‐β signaling is protective for fibrosis and hypertrophic remodeling (Ikeuchi et al., 2004).

Here, we demonstrate, as with the aging heart, there is an association between the level of myocardial TGF‐β ligand expression and the level of myocardial senescence. It is, therefore, possible that the SASP also contributes to driving fibrosis and scar formation; a hypothesis supported by the reduced scar size observed in the navitoclax‐treated animals. While our observations initially appear at odds with studies demonstrating that senescence is required to attenuate cardiac fibrosis (Meyer et al., 2016; F. L. Zhu et al., 2013), it is important to note that navitoclax does not inhibit senescence, but induces apoptosis once senescence is achieved (Y. Zhu et al., 2015). Therefore, the two observations are entirely compatible. Indeed Zhu *et al* propose that while fibroblast senescence reduces collagen deposition in the short term post‐MI, senescent fibroblasts are also a source of chronic inflammation contributing to ongoing cardiac fibrosis in the longer term (F. L. Zhu et al., 2013).

A limitation of the current treatment strategy arises as we did not precisely characterize the kinetics of senescence accumulation following IR nor the dynamics of senescent cell elimination following navitoclax treatment. Exhaustive studies characterizing kinetics of accumulation and elimination may allow for an optimized treatment regime with the potential to further improve outcome.

In the absence of MI, aged animals demonstrated an increase in CM renewal following either pharmacogenetic or pharmacological clearance of senescence (Anderson et al., 2019; Lewis‐McDougall et al., 2019). However, senescence elimination following IR did not enhance CM regeneration in the young animals used in our study. Following MI, both the vehicle and navitoclax‐treated mice displayed a similar CM regenerative response, corresponding to a renewal of 0.47% and 0.38% CMs, respectively, during the first week following MI. These rates are consistent with those previously reported for MI hearts without therapeutic intervention (Malliaras et al., 2013), suggesting that young animals have a CM regenerative potential that is not impeded by senescence. In contrast, we noted an increase in both endothelial proliferation and vessel density in the hearts of navitoclax‐treated mice. Based on our *in vivo* studies demonstrating an increase in peri‐infarct vascularization in the navitoclax‐treated animals, the observed association between post‐IR senescence and the expression of anti‐angiogenic cytokines including IP‐10, together with the known anti‐angiogenic properties of some SASP components (Coppe et al., 2010) it is possible that following IR senescent cells attenuate angiogenesis directly as a result of the expression of an anti‐angiogenic SASP. As such, the increased vascularization post‐IR following navitoclax treatment may occur because of a reduction in the SASP. However, it is also possible that senescence and the SASP contribute to attenuated angiogenesis as a result of the bystander effect and the induction of senescence in the endothelial cell population reducing proliferative potential as a result of cell‐cycle arrest, a hypothesis supported by our *in vitro* conditioned media studies. Additional *in vivo* experiments quantifying senescence specifically in endothelial populations would be required to dissect these two possible mechanisms. Regardless of the mechanisms involved, improved angiogenesis and improved myocardial oxygenation may also contribute to the observed improvement in cellular respiration. In this context, however, we have previously demonstrated that myocardial aging and accumulated senescence is associated with mitochondrial dysfunction (Anderson et al., 2019). Therefore, a reduction in senescence cells with dysfunctional mitochondria, by navitoclax treatment, may also contribute directly to a global increase in mitochondrial function.

In conclusion, these data suggest a key role of myocardial senescence signaling downstream of the initial oxidative stress response to reperfusion. We suggest that targeting senescence is a valid and clinically feasible strategy to attenuate maladaptive remodeling and promote recovery post‐IR. Major advantages of senolytic treatment over current strategies include 1) targeting senescence which occurs as a result of the cellular stress associated with IRI provides an extended therapeutic window; and 2) targeting senescence attenuates multiple components of the inflammatory responses subsequent to IR, which are detrimental to recovery (Prabhu & Frangogiannis, 2016; Sánchez‐Hernández et al., 2020). Recent studies have begun to trial senolytics in patients suffering from pulmonary fibrosis or kidney disease (Hickson et al., 2019; Justice et al., 2019), and if senolytics prove to be effective and safe, they could be transformative for cardiovascular medicine.

## 4 EXPERIMENTAL PROCEDURES

### 4.1 Mouse model and myocardial infarction with reperfusion

Male C57BL/6 J mice at 3‐4 months of age were used in all studies. Intra‐operative analgesia was induced by pre‐treating mice with fentanyl/fluanisone (0.4 ml/kg, Hypnorm), prior to anesthesia using isoflurane, which was maintained using mechanical ventilation following endotracheal intubation (3% isoflurane/97% oxygen, 130‐140 stroke rate, stroke volume initially 5 ml/kg—increased to 7.5 ml/kg post‐thoracotomy). At the fourth‐intercostal space, left‐side thoracotomy was executed to allow partial removal the pericardium and enable a 7‐0 prolene suture to be placed around the left anterior descending artery (LAD) and loosely tied. An infarction was induced by inserting 2 mm PE‐10 tubing into the suture loop and tightening the suture knot to terminate blood flow for 60 minutes. The tubing was removed to allow myocardial reperfusion, the chest cavity closed, and 0.05 mg/kg Vetergesic was provided as analgesia. Naïve mice (no surgical intervention) were used as control. All animal studies were conducted in accordance with the Guidance on the Operation of the Animals (Scientific Procedures) Act, 1986 (UK Home Office), and approved by the local ethics committee.

### 4.2 In vivo navitoclax (ABT263) treatment

Navitoclax was prepared in a lipid vehicle solution consisting of EtOH, polyethylene glycol 400 and Phosal 50 PG in a 1:3:6 ratio, respectively (Chang et al., 2016). Mice were randomly assigned to experimental groups. Navitoclax (50 mg/kg/day via oral gavage) was provided for the dose timing regimes detailed in the main text. When required, EdU (100 mg/kg/d) was provided via intraperitoneal injection (Richardson, 2016; Richardson et al., 2015).

### 4.3 Histology and Immunohistochemistry

Immunohistochemistry was performed as described previously (Richardson et al., 2012). 10 μm sections were used for all studies. Primary antibodies used: rat ant‐p21 (HUGO291, Abcam ab107099), goat‐anti‐troponin C (Abcam, ab30807), rabbit anti‐p16 (Rockland, 100‐401‐170) and rat anti‐CD31 (MEC13.3, BD Biosciences, 550274). Secondary antibodies used were donkey anti‐rat AF594 (Life Technologies, A21209), donkey anti‐goat AF 488 nm (Life Technologies, A11055), donkey anti‐rabbit AF594 (Life Technologies, R37119), donkey anti‐goat AF488 (Life Technologies, A11055) and donkey anti‐mouse AF647 (A31571, Life Technologies). Slides were mounted in Vectarshield containing DAPI (Sigma, MBD0015). 5‐ethynyl‐2‐deoxyuridine (EdU) labeling performed with Invitrogen Click‐iT EdU Alexa Fluor 594 Imaging Kit (Life Technologies, C10339).

### 4.4 Rockland P16 antibody specificity assessment

To assess the specificity of the rabbit anti‐p16 (Rockland, 100‐401‐170) we compared p16^Ink4a^ antibody staining (Rockland, 100‐401‐170) to p16^**Ink4a**^ mRNA expression using RNAscope^TM^ (Mm‐Cdkn2a‐tv2, 447491, ACD, C1 probe). Staining was performed in the hearts of transgenic mice which allow for a CM specific knock‐out of p16^Ink4a^ exon1α and control littermates with wild‐type (WT) p16^Ink4a^ expression, at 5 weeks post‐IR (Figure S3). The α‐Myosin Heavy Chain‐MerCreMer transgenic line (MerCreMer) (Sohal et al., 2001) and a transgenic mouse in which exon1α of p16^Ink4a^ is flanked by loxp sites (Sharpless et al., 2001) (p16^f/f^) were crossed to generate MerCreMer^+/−^:: p16^f/f^ **,** which when provided tamoxifen excise exon 1α of p16^inka4^ specifically the α‐myosin heavy chain‐expressing CM population and WT:: p16^f/f^ littermate controls. Genotype was confirmed using the primers for Cre F‐TAACCAGTGAAACAGCATTGCTG, Cre R‐ GGACATGTTCAGGGATCGCCAGGCG, p16^Ink4a^ floxed‐GTATGCTATACGAAGTTATTAGGTACTGC. p16^Ink4a^ wild‐type GTTTTGGAGCAGCAGGGATT and p16^Ink4a^ common‐CTATGTCAGATTTGGCTAGGGAGT. Mice were treated with 4‐OH‐tamoxifen (Sigma) dissolved in peanut oil at a concentration of 0.5 mg/day (I.P) for 2 weeks at previously described (Malliaras et al., 2013) and subjected to cardiac IR (Figure S3A). Excision of the p16^Ink4a^ exon 1α was verified by PCR,

using LCred primers, specific to sequences located outside the loxP sites that produce a PCR product of approximately 270 bp or an unamplifiable product of 3.9 kb. p16^Ink4a^ LCred F‐TACCACAGTTTGAACAGCGTGA and p16^Ink4a^ LCred R‐AACCAACTTCCTCCTTCCCC (Figure S3B). Immunohistochemistry was performed using the rabbit anti‐p16 (Rockland, 100‐401‐170) and donkey anti‐rabbit AF594 (Life Technologies, R37119). RNAscope^TM^ was performed according to the manufacturer's protocol (RNAscope^TM^ Multiplex Fluorescent Kit v2 User Manual, 323100‐USM, ACD) (Advanced Cell Diagnostics, 2018). For both staining techniques sections were co‐stained with trop‐c (anti‐troponin C, 1:800, ab30870, Abcam) and DAPI (1:500, MBD0015, Sigma). Sections mounted in Dako Fluorescent Mounting Medium (S3023, Agilent) (Figure S3C,D).

### 4.5 Senescence‐associated β‐galactosidase staining

Cryo‐embedded sections were stained using the senescence‐associated β‐galactosidase (SA‐β‐Gal) staining kit (Cell Signaling Technology, 9860) as per the manufacturer's instructions with the following modifications for tissue sections. Slides were thawed at room temperature, fixed using the provided fixative solution for 15 minutes, and washed three times in PBS. The β‐galactosidase staining solution was prepared at pH 6 and added to each slide. Slides were incubated at 37°C for 24 hours. Once developed slides were washed with PBS, dehydrated in 95% and 100% ethanol solutions, washed in Histoclear, and mounted with Histomount.

### 4.6 Real‐Time PCR (qRT‐PCR)

Qiagen RNA extraction kit was used for RNA isolation. First‐strand cDNA synthesis kit (Thermo Fisher Scientific, Waltham, MA, US) was used for cDNA synthesis. Real‐time PCR was performed in a 7500 Fast Real‐Time PCR System using Taman probes (Life Technologies Ltd, UK) GAPDH (Mm99999915_g1), p21 (Rasa3) (Mm00436272_m1) and p16 (Cdkn2a) (Mm00494449_m1). Data presented normalized to GAPDH.

### 4.7 Immuno‐FISH

Telomere‐associated DNA damage foci (TAF) were detected by performing Immuno‐FISH, as previously described (Passos et al., 2007), on cryo‐embedded heart sections. Briefly, sections that were labeled with rabbit monoclonal anti‐γH2Ax (20E3, Cell Signaling Technology, 9718) and following secondary labeling with goat‐anti‐rabbit IgG biotinylated (VectorLab, PK‐6101), sections were fixed with methanol: acetic acid (3:1), dehydrated and incubated with PNA hybridization mix with 5% blocking reagent (Roche) containing 2.5 μg/ml Cy3‐labeled telomere‐specific (CCCTAA) peptide nucleic acid probe (Panagene).

### 4.8 Microscopy, image analysis, and quantification

All images were acquired using Axio Imager (Zeiss) and analyzed using ZEN 2.3 (Zeiss). All quantifications were performed blinded to treatment and genotype using digital image analysis (ImageJ; U.S. National Institutes of Health; http://rsbweb.nih.gov/ij/). Quantification of p16, p21, EdU was performed as we described previously (Anderson et al., 2019) except that quantification was restricted to the peri‐infarct region; defined as the region proximal to the infarct (Figure S1A, red region). A minimum of 20 images/heart over 5 individual sections were taken within this peri‐infarct area (total approx. 6000 cells analyzed per heart). For each image, the total number of CMs (identified by trop‐c expression) and the number of CMs expressing the protein of interest were quantified, allowing the percentage CM expressing each protein to be calculated. Similarly, the number of interstitial cells (identified as DAPI^+^ trop‐c^−^) expressing the protein of interest was quantified allowing the percentage expression to be calculated. For CM EdU quantification, WGA was also used to allow the identification of individual CMs. For endothelial EdU quantification, the total number of CD31^+^ endothelial cells which had incorporated EdU was quantified in each image. CM hypertrophy was quantified as described previously (Correia‐Melo et al., 2019), following staining with the membrane marker wheat germ agglutinin (WGA) (W32466, Invitrogen, UK).

### 4.9 Magnetic resonance imaging and analysis

Magnetic resonance images were acquired using a horizontal bore 7.0 T Varian microimaging system (Varian Inc., Palo Alto, CA, USA) equipped with a 12‐cm microimaging gradient insert (40 gauss/cm) and analyzed as described previously (Redgrave et al., 2017).

### 4.10 Liquid Chromatography Mass Spectrometry (LC‐MS/MS) with SWATH acquisition (Sequential Windowed Acquisition of All Theoretical Fragment Ion Mass Spectra)

#### 4.10.1 Left ventricular sample preparation

At the time‐points detailed in the results section, hearts were collected, and the left ventricle (LV) was dissected apical to the suture and placed in RIPA buffer (R0278, Sigma) containing protease inhibitors. Tissue was homogenized and proteins precipitated with acetone. Protein was dissolved in 8 M urea, 10 mM HEPES at pH 8.0. 200 µg of protein was reduced with 30 mM DTT at 30°C for 30 minutes followed by alkylation with 10 mM Iodoacetamide. Urea molarity was reduced to 1.5 M prior to trypsin digestion (Worthington, TPCK treated) at a ratio of 20:1 (protein: trypsin). The digestion was stopped with trifluroacetic acid (TFA) and proteins purified with a self‐packed C18 stage (Rappsilber et al., 2007). Peptides were eluted, dissolved in 3% acetonitrile with 1% TFA, and sonicated.

### 4.11 Nano‐LC‐MS/MS

Analysis was performed with an AB‐Sciex 6600‐Tripletof operating in SWATH mode. Protein digests were injected into a mass spectrometer through an UltiMate 3000 RSLCnano system. Samples were loaded onto a 300 μm x 5 mm C18 PepMap C18 trap cartridge in 0.1% formic acid and then separated at 300 nl/min using a 95 min nonlinear gradient (3‐30%ACN:87 min; 30‐40%:10 min; 40‐90%:5 min) using a 75 μm x 25 cm C18 column (ReproSil‐Pur Basic‐C18‐HD, 3 µm, Dr. Maisch GmbH). The eluent was directed to an Ab‐Sciex TripleTOF 6600 mass spectrometer through the AB‐Sciex Nano‐Spray 3 source, fitted with a New Objective FS360‐20‐10 emitter. SWATH acquisition was performed in a mass range of 400‐800 m/z, with 75 variable SWATH bins and accumulation time of 40 milliseconds. The number of SWATH bins was estimated based on a pilot DDA run of a random sample using SWATH variable window calculator ver.1.1 (AB Sciex).

All the raw data with the associated search results were deposited in a publicly accessible repository (https://massive.ucsd.edu/, MSV000085040) (Wang et al., 2018).

### 4.12 Data analysis

The SWATH files were processed in PeakView 2.1 (AB Sciex) with the SWATH MSMSall micro app (top 1000 peptides/protein, top 5 transitions/peptide, confidence threshold: 95%, FDR threshold: 1%, modified peptides excluded, extraction window: 10 min, XIC width: 10 ppm). The mouse heart spectral library was obtained from ProteomeXchange (Vizcaíno et al., 2014) (PXD017795, MSV000085036) and pre‐aligned to a randomly selected SWATH file, as described (Palmowski et al., 2019). The results were exported, via MarkerView 1.2.1 (AB Sciex), as tab‐separated ASCII files and processed using the Perseus software framework (Tyanova et al., 2016). Protein peak areas were transformed to log scale, followed by median subtraction (sample median was subtracted to account for loading differences), and the values derived from technical replicates were averaged. Individual proteins had to be quantifiable in all samples to meet the criteria for further analysis. T‐test was applied to identify proteins that differed in relative abundance between experimental groups and permutation‐based false discovery rate was estimated to account for multiple comparisons. Protein groups, following the relative abundance profiles of interest, were then extracted. The log_2_ peak areas were first z‐scored for each protein and the reference profile was defined. For each protein, a sum of squared differences (differences between measured values and the reference) was calculated as a measure of distance from the reference. The resulting values were then used for permutation‐based FDR calculation (R‐Script file supplementary data) to allow confident identification of proteins that followed a predefined abundance profile. Association with particular subcellular compartments, biological functions or pathways, was assessed for molecules differentially abundant between conditions and/or sharing the same abundance profile, using STRING v11.0 (Szklarczyk et al., 2019), followed by manual interrogation. Statistical background for pathway/enrichment analysis was restricted only to proteins detected in the analyzed samples.

### 4.13 Scar size quantification

Masson's trichrome staining was performed in order to visualize scar tissue (Bogatyryov et al., 2013). Hearts were sectioned into 5 sets of slides, 10 slides per set and stained with Masson's trichrome. Each set of slides was imaged and analyzed using the Leica Digital Image Hub. The LV area was calculated by measuring the epicardial area and subtracting the endocardial area. The infarct area was then measured and the percentage of LV that is infarct calculated to analyze scar size.

### 4.14 Cell culture

Cell culture work has been performed using human embryonic lung MRC5 fibroblasts acquired from European Collection of Authenticated Cell Cultures (05072101), Salisbury, UK, and mouse cardiac endothelial cells from Cedarlane (CLU510‐P).

### 4.15 Statistical analysis

All analyses were performed in a blinded manner. All analyses were performed using GraphPad Prism 8.0. Data were first tested for normality. Parametric data were analyzed using a 2‐tailed t‐test or one‐way ANOVA, as appropriate.

## 5 FUNDING STATEMENT

This study was funded by The British Heart Foundation grants; PG/19/15/34269, PG/14/86/31177, PG/18/25/33587, and PG/18/57/33941. The Wellcome Trust; and the Newcastle Healthcare Charity. JFP would like to acknowledge the Ted Nash Long Life Foundation.

## 6 PERMISSION TO REPRODUCE MATERIAL FROM OTHER SOURCES

Figures created with BioRender.com with paid subscription.

## CONFLICT OF INTEREST

None.

## AUTHOR CONTRIBUTIONS

ED, AW, RR, PP, ST‐C, AS, JC, EJ, LDS, EG, OEY, and YS performed experiments. GDR, JFP, JMP, HMA, IS, WAO, MT, and DG contributed to supervision. PP, MT, and JFP designed and supervised aspects of the study. GDR designed, supervised, and oversaw the study and wrote the manuscript with input from all authors.

## Supporting information

Figures S1–S9
Table S1

Dataset S1

Dataset S2

Supplementary Material

## Data Availability

The data with the associated search results are deposited in a publicly accessible repository (https://massive.ucsd.edu/, MSV000085040).
