# Supplementary material for: Clearance of senescent cells during cardiac ischemia–reperfusion injury improves recovery
Source: Aging Cell. 2020 Sep 29;19(10):e13249. doi: 10.1111/acel.13249 (PMC7576252; doi:10.1111/acel.13249)
Supplement: Supplementary file 1 — Figures S1–S9 Table S1 [file ACEL-19-e13249-s001.docx]

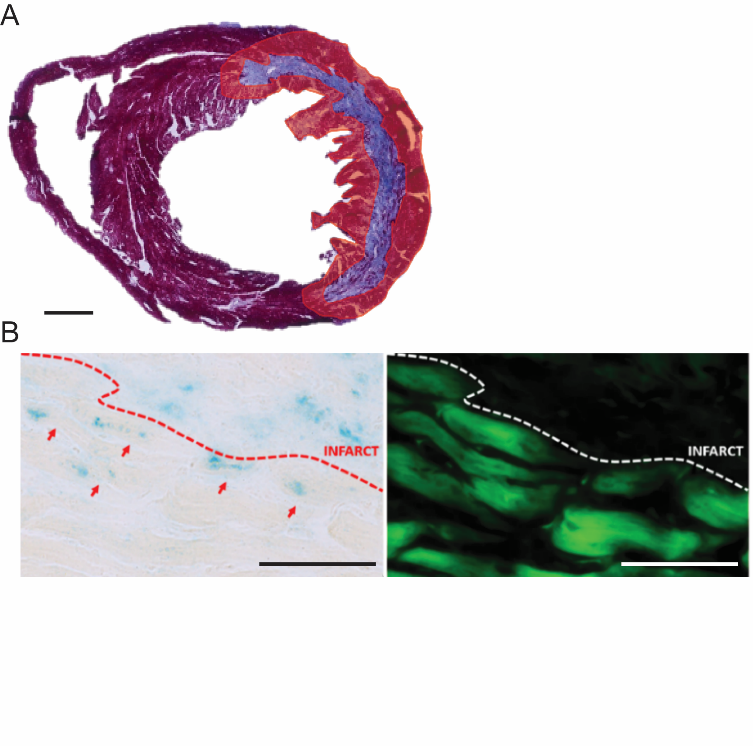


**Supplementary Figure 1.** **A)** The defined peri-infarct region used for quantification studies. Scale bar 500µm. **B)** Representative image of CMs staining positive for SA-β-Gal in the peri-infarct region following IR (blue – SA-β-Gal; green – Autofluorescence). Scale bar 50µm.

**
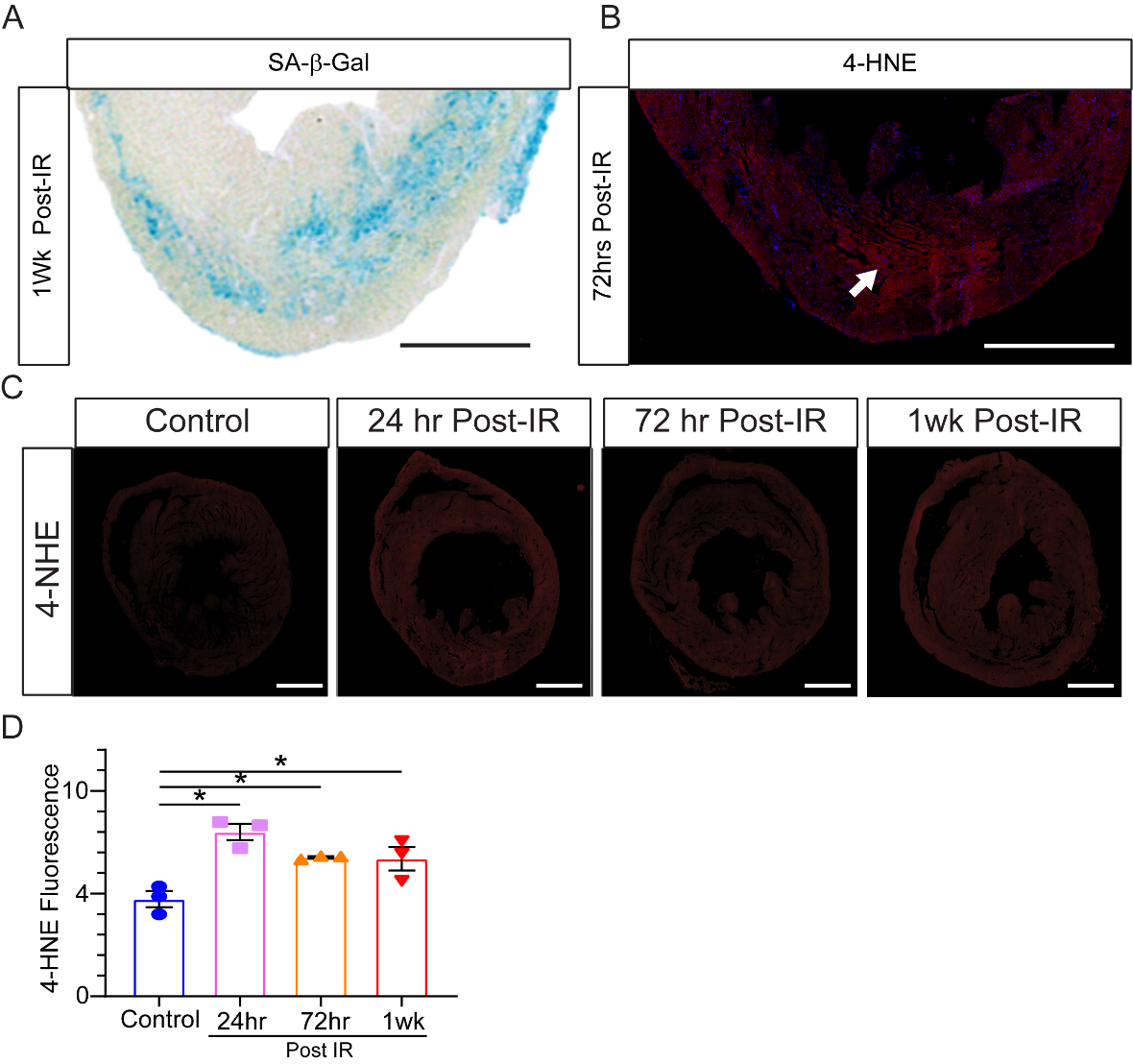
**

**Supplementary Figure 2.** **A and B)** Representative image of 4-HNE and SA-β-Gal staining at 72-hours and 1-week, respectively. White arrow indicates the area of high 4-HNE in the area of infarct. **C)** Representative image of 4-HNE at each investigated time point post-IR. **D)** Quantification of 4-HNE demonstrates increased myocardial superoxide production in the heart subjected to 60 minute LAD-ligation with reperfusion. N=3/group. All scale bars 2mm. Data are mean±SEM, analysis by one-way ANOVA followed by Tukey’s post hoc test, * P<0.05.


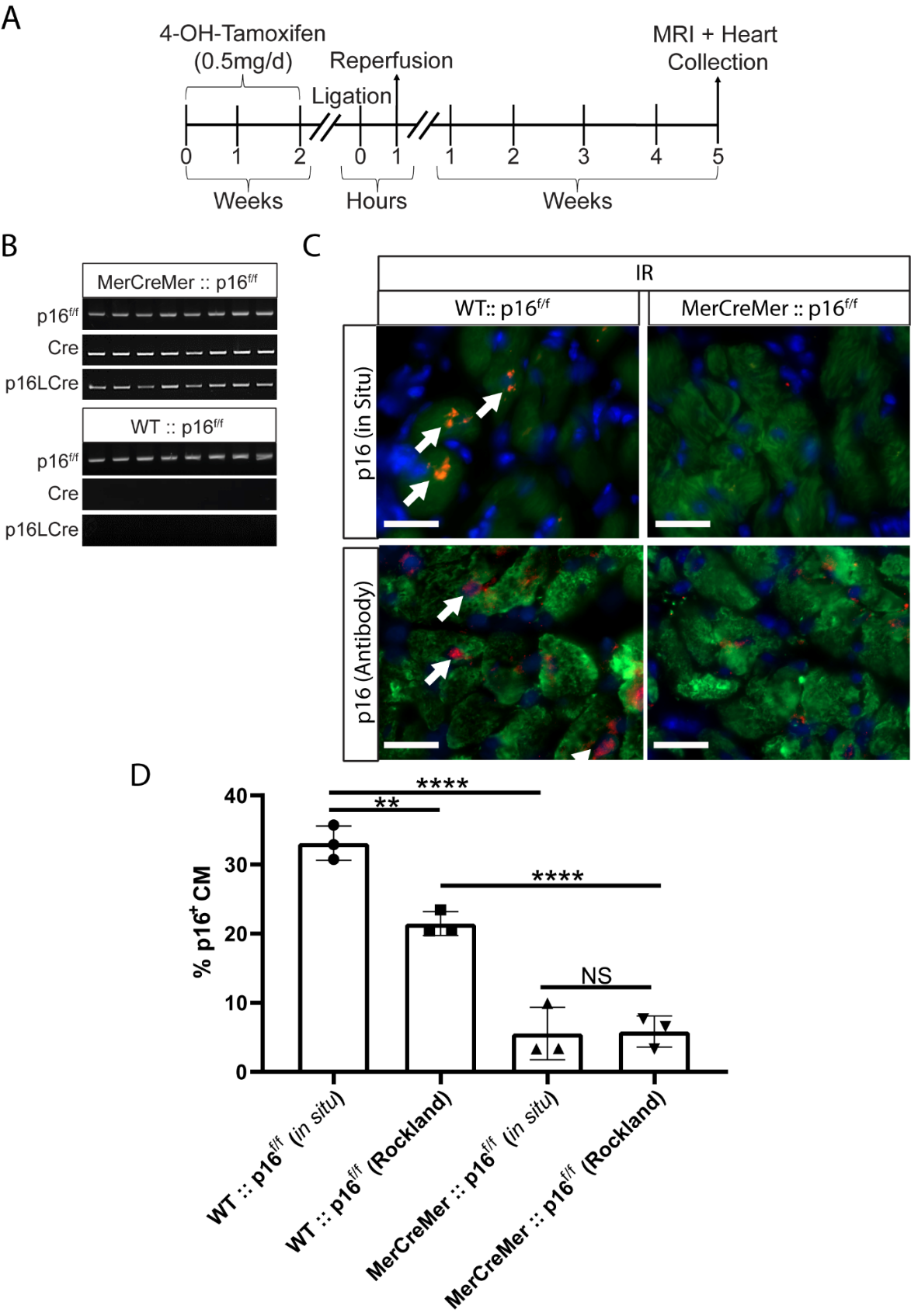


**Supplementary Figure 3. Assay of Rockland p16 antibody specificity. A and B)** Experimental design and validation of the transgenic model. Mouse genotypes were established using primers specific to the floxed p16^inka4^ allelle (p16^f/f^) and MercreMer (cre). Bitransgenic MerCreMer-p16^f/f^ mice and WT-p16^f/f^ control mice were given 4-OH-tamoxifen for 14 days. Cre-mediated excision of exon 1α from the MerCreMer :: p16^f/f^ mice was confirmed by PCR using p16LCre primers which span the excised region. All mice were subjected to IR and hearts collected 5 weeks post-IR. **C)** Representative images of p16 expression as assessed by *in situ* hybridization or using the Rockland antibody at 5 weeks post-IR. Arrows indicate p16**^+^** CMs identified by trop-c expression (p16 red and trop-c green). Scale bars 20µm. **D)** Quantification of the percentage of p16 expressing CM in the peri-infarct region for each assay and genotype. Data suggests the antibody has specificity for murine p16 protein, as there are is comparable staining between both assays. In addition, antibody specificity is indicated by the significant reduction in CM antibody labelling in the knock-out model, a reduction comparable to that observed by *in situ* hybridization. N=3/group. Data are mean±SEM, analysis by one-way ANOVA followed by Tukey’s post hoc test ****P<0.0001 **P<0.01.


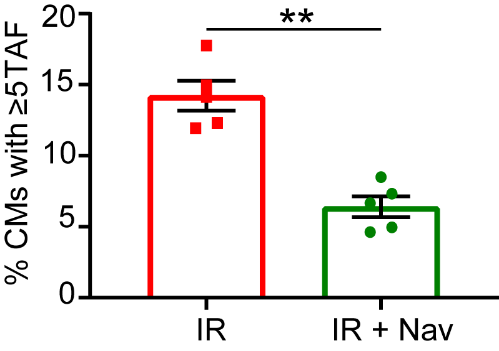


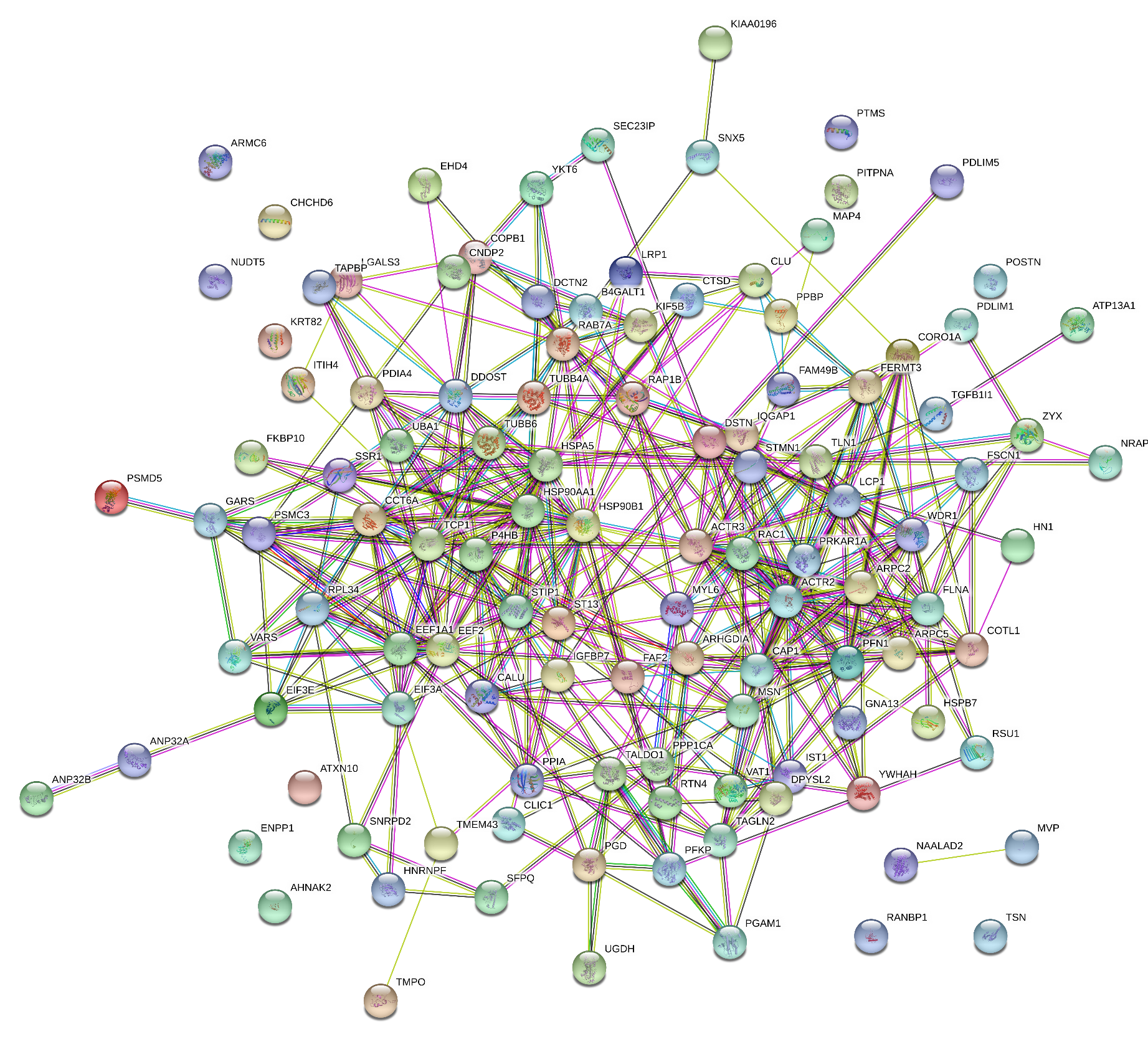
**Supplementary Figure 4.** Quantification of the percentage of ≥5 TAF^+^ CM in the peri-infarct region of the left ventricle peri-infarct region in vehicle or navitoclax treated mice at 5 weeks post-IR. N=5/group. Data are mean±SEM, analysis by 2-tailed unpaired t-test, ** P<0.01.


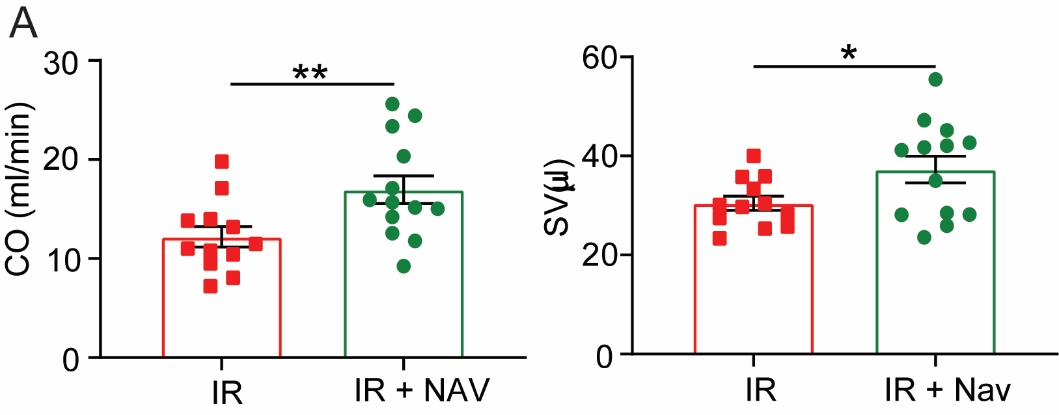


**Supplementary Figure 5.** Data obtained from MRI analysis for mice treated with either vehicle or navitoclax post-IR. IR N=12 and IR+Nav N=13. Cardiac output (CO ml/min) and stroke volume (SV μl). Data are mean±SEM, analysis by 2-tailed unpaired t-test **P<0.01.

**
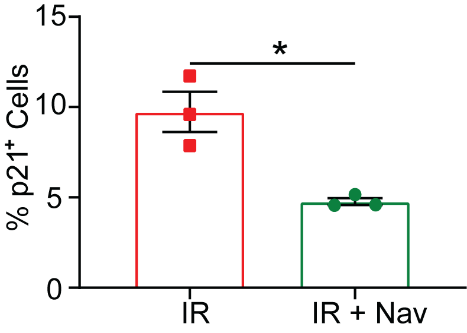
**

**Supplementary Figure 6.** Quantification of the percentage of p21 expressing cells in the LV peri-infarct region of vehicle control and navitoclax treated animals at 7 days post-IR. N>3/group. Data are mean±SEM, analysis by 2-tailed unpaired t-test *P<0.05.


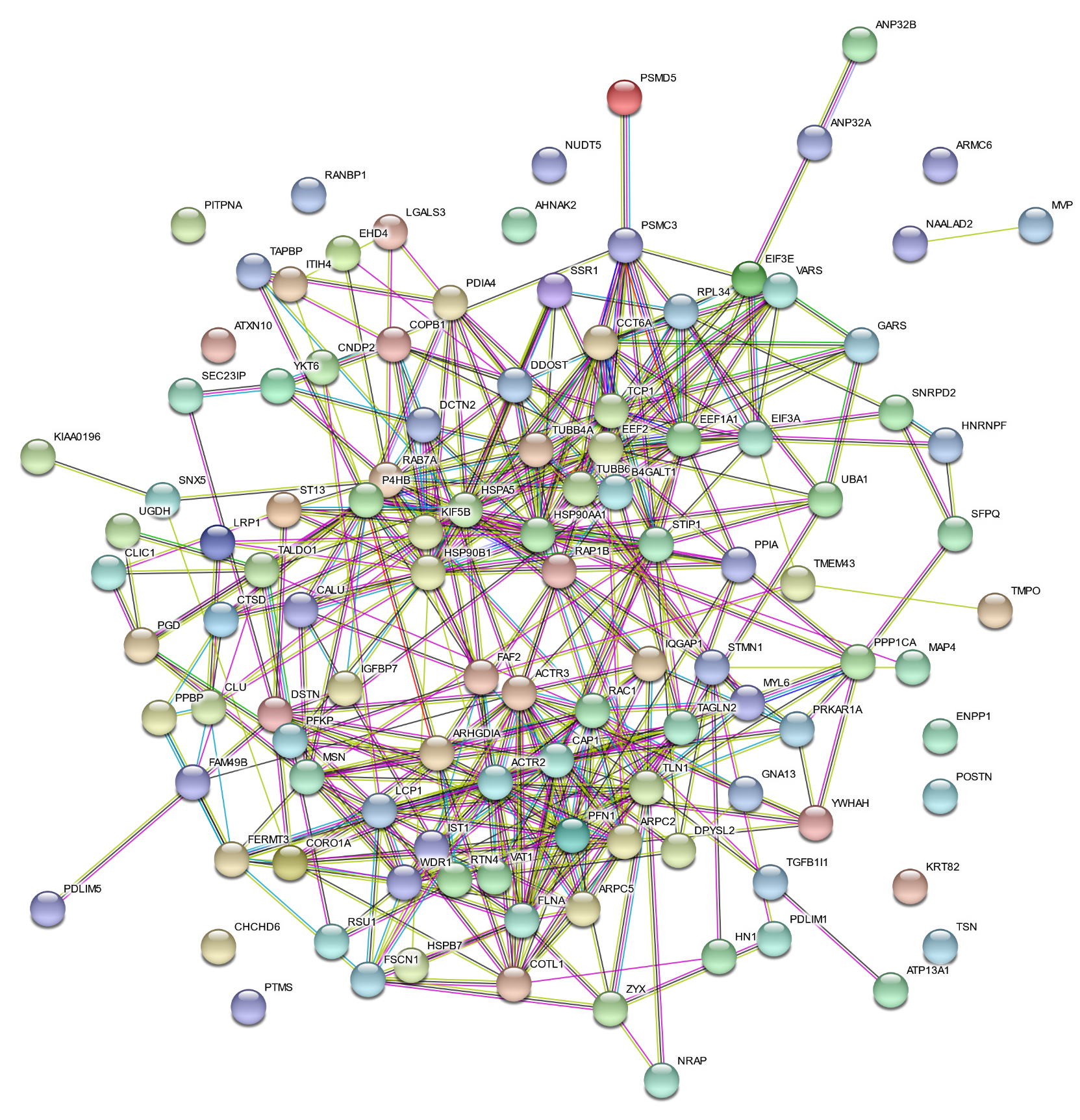


**Supplementary Figure 7.** Protein network for proteins identified for expression profile 1 (increased following IR and reduced after navitoclax treatment).


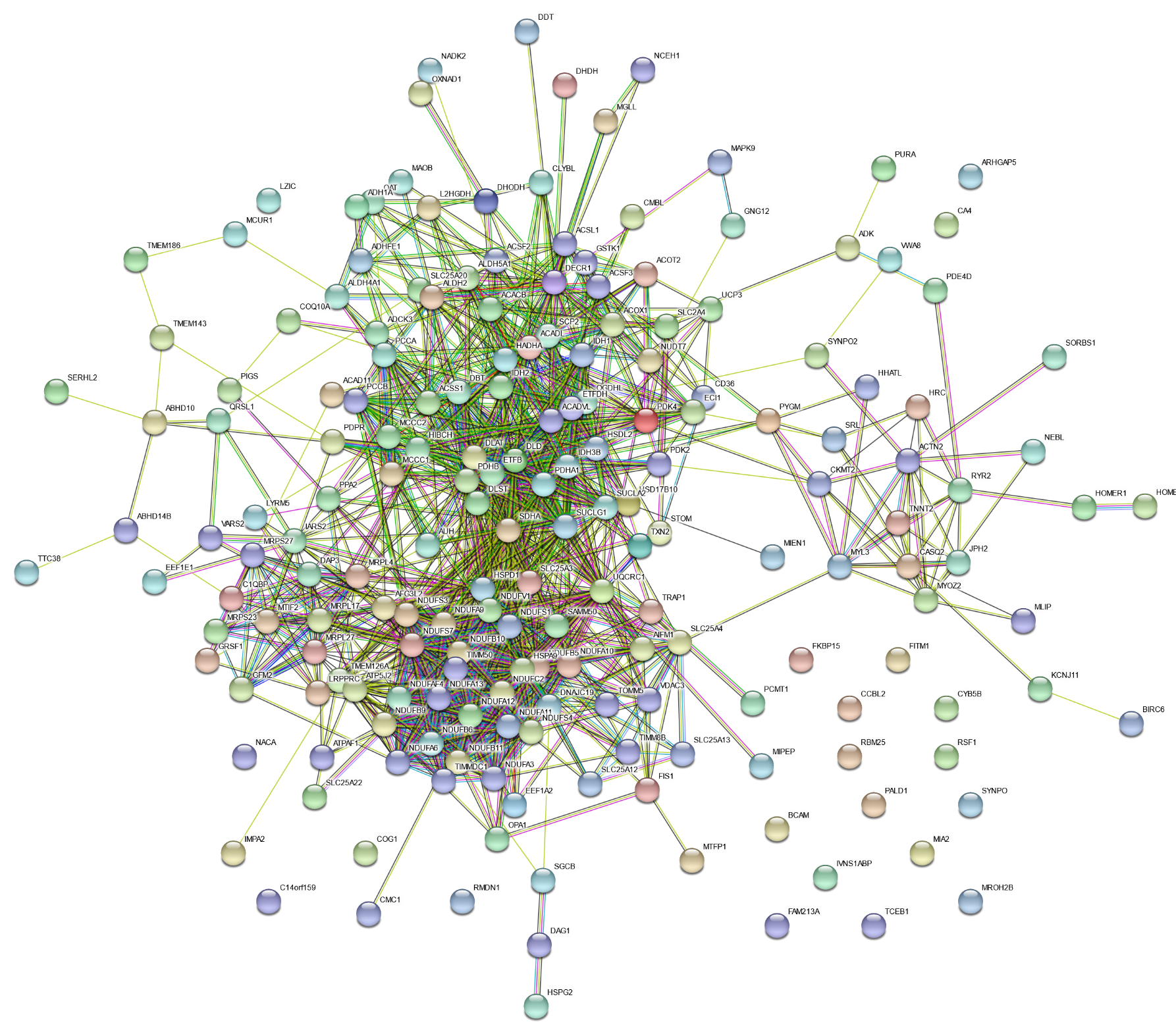


**Supplementary Figure 8.** Protein network for proteins identified for expression profile 2 (decreased following IR and rescued by navitoclax treatment).

**
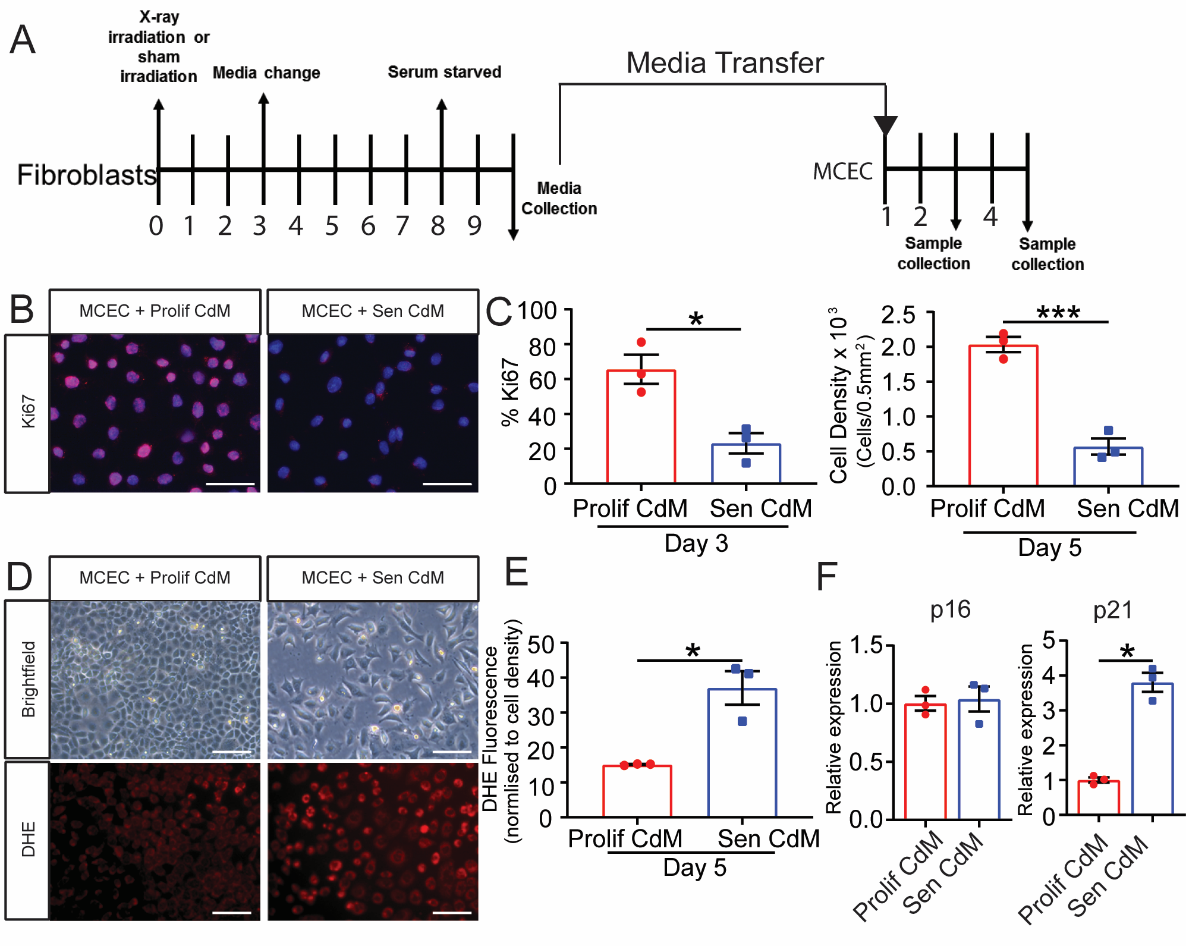
Supplementary Figure 9. SASP reduces proliferation in primary cardiac endothelial cells. A)** Experimental Design. MRC5 fibroblasts were either induced to senescence with X-ray irradiation or sham irradiated. Following 10 days, conditioned medium (CdM) was collected and used to culture mouse cardiac endothelial cells (MCEC). Treated endothelial cells were collected on day 3 and day 5. **B)** Representative images of Ki67 expression in MCEC cultures on day 3. **C)** Quantification of the percentage of MCEC expressing Ki67 at 3 days and MCEC density at 5 days following culture in CdM from either proliferative (Prolif CdM) or senescent fibroblasts (Sen CdM). **D)** Representative bright-field images and images of DHE staining of MCECs at day 5 for each CdM. **E)** Quantification of the in the intensity of DHE fluorescence normalized to total cell number at 5 days of CdM treatment. **F)** Real-time PCR gene expression analysis for relative p16 and p21 expression (normalized to GAPDH). All scale bars 50µm. Analysis by 2-tailed unpaired t-test, *P<0.05, **P<0.01, ***P<0.001.

**Supplementary Table 1. Values from MD-44 and TGFB1-3 Arrays.** Data from n=3. Statistical test by one-way ANOVA.

| **Cytokine** | **Concentration (pg/ml ± SD)** | | | **p Value** | | |
| --- | --- | --- | --- | --- | --- | --- |
|  | **Control** | **IR + Veh** | **IR + Nav** | **Control**  **vs**  **IR + Veh** | **Control**  **vs**  **IR + Nav** | **IR + Veh**  **vs**  **IR + Nav** |
| Eotaxin | 6.74 ± 2.67 | 16.10 ± 4.28 | 8.87 ± 2.29 | 0.0270 (*) | 0.7081 | 0.0728 |
| EPO | - | - | - | - | - | - |
| Fractalkine | 71.21 ± 2.53 | 90.27 ± 4.56 | 67.98 ± 4.49 | 0.0022 (**) | 0.3579 | 0.0014 (**) |
| G-CSF | 0.78 ± 0.17 | 1.17 ± 0.38 | 0.56 ± 0.07 | 0.1981 | 0.5537 | 0.0499 (*) |
| GM-CSF | - | - | - | - | - | - |
| IFNB-1 | 66.52 ± 1.94 | 67.22 ± 1.05 | 60.25 ± 2.88 | 0.9130 | 0.0243 (*) | 0.0153 (*) |
| IFNγ | 0.95 ± 0.79 | 2.09 ± 0.13 | 0.62 ± 0.72 | 0.2532 | 0.8323 | 0.1373 |
| IL-1α | 51.00 ± 6.92 | 17.17 ± 5.02 | 14.14 ± 2.21 | 0.0005 (***) | 0.0003 (***) | 0.7571 |
| IL-1β | 7.20 ± 0.77 | 6.65 ± 1.10 | 5.28 ± 0.36 | 0.6952 | 0.0560 | 0.1619 |
| IL-2 | 25.73 ± 0.52 | 19.78 ± 2.93 | 17.75 ± 1.41 | 0.0200 (*) | 0.0051 (**) | 0.4403 |
| IL-3 | 0.94 ± 0.12 | 0.84 ± 0.10 | 0.67 ± 0.16 | 0.6607 | 0.0969 | 0.3003 |
| IL-4 | 0.19 ± 0.01 | 0.15 ± 0.01 | 0.12 ± 0.05 | 0.2437 | 0.1013 | >0.9999 |
| IL-5 | 0.01 ± 0.00 | 0.05 ± 0.00 | 0.01 ± 0.00 | - | - | - |
| IL-6 | 1.21 ± 0.04 | 2.73 ± 0.64 | 1.53 ± 0.71 | 0.0347 (*) | 0.7631 | 0.0841 |
| IL-7 | 3.57 ± 0.46 | 2.93 ± 1.02 | 1.74 ± 0.42 | 0.5349 | 0.0403 (*) | 0.1659 |
| IL-9 | 229.7 ± 6.34 | 185.2 ± 5.28 | 191.8 ± 6.98 | 0.0003 (***) | 0.0007 (***) | 0.4525 |
| IL-10 | 12.72 ± 0.60 | 9.56 ± 2.14 | 8.57 ± 0.80 | 0.0669 | 0.0229 (*) | 0.6663 |
| IL-11 | 0.89 ± 0.90 | 2.76 ± 0.76 | 0.60 ± 0.38 | 0.0366 (*) | 0.6412 | 0.0301 (*) |
| IL-12/p40 | 4.94 ± 1.17 | 3.77 ± 1.87 | 4.04 ± 1.11 | 0.6013 | 0.7295 | 0.9722 |
| IL-12/p70 | 3.34 ± 0.59 | 3.54 ± 1.84 | 3.16 ± 0.98 | 0.9798 | 0.9831 | 0.9286 |
| IL-13 | - | - | - | - | - | - |
| IL-15 | 33.18 ± 2.24 | 27.81 ± 4.00 | 19.19 ± 2.23 | 0.1437 | 0.0027 (**) | 0.0268 (*) |
| IL-16 | 241.05 ± 61.39 | 382.7 ± 53.75 | 216.31 ± 32.86 | 0.0328 (*) | 0.8270 | 0.0165 (*) |
| IL-17 | 0.13 ± 0.10 | 0.14 ± 0.12 | 0.03 ± 0.01 | 0.9989 | 0.3746 | 0.3554 |
| IL-20 | - | - | - | - | - | - |
| IP-10 | 5.31 ± 0.69 | 48.57 ± 27.52 | 8.26 ± 2.97 | 0.0370 (*) | 0.9723 | 0.0488(*) |
| KC | 7.32 ± 2.72 | 13.73 ± 10.69 | 3.94 ± 1.01 | 0.4756 | 0.7970 | 0.2207 |
| LIF | 0.66 ± 0.18 | 1.58 ± 0.54 | 0.77 ± 0.09 | 0.0760 | >0.9999 | 0.2209 |
| LIX | 268.7 ± 27.41 | 229.2 ± 19.23 | 208.8 ± 36.03 | 0.6097 | 0.1840 | >0.9999 |
| MCP-1 | 3.81 ± 0.86 | 11.39 ± 9.57 | 1.49 ± 1.14 | 0.4260 | 0.9107 | 0.2105 |
| MCP-5 | 23.72 ± 7.91 | 224.4 ± 148.2 | 48.53 ± 16.07 | 0.0655 | 0.9345 | 0.1022 |
| M-CSF | 0.84 ± 0.20 | 0.85 ± 0.69 | 0.52 ± 0.10 | 0.9996 | 0.7363 | 0.7230 |
| CCL22 | 0.99 ± 0.23 | 5.03 ± 0.49 | 2.86 ± 0.44 | <0.0001 (****) | 0.0031 (**) | 0.0014 (**) |
| MIG | 36.13 ± 15.11 | 155.8 ± 100.7 | 52.99 ± 25.51 | 0.1134 | 0.9387 | 0.1751 |
| MIP-1a | 52.55 ± 9.31 | 36.41 ± 7.62 | 34.19 ± 7.56 | 0.1219 | 0.0800 | 0.9421 |
| MIP-1β | - | - | - | - | - | - |
| MIP-2 | 273.1 ± 31.23 | 244.4 ± 10.57 | 214.0 ± 11.89 | 0.2675 | 0.0272 (*) | 0.2366 |
| MIP-3α | 1.47 ± 0.06 | 1.05 ± 0.19 | 0.95 ± 0.02 | 0.0089 (**) | 0.0030 (**) | 0.5380 |
| MIP-3β | 31.22 ± 12.74 | 134.18 ± 16.86 | 79.63 ± 16.55 | 0.0005 (***) | 0.0204 (*) | 0.0119 (*) |
| RANTES | 2.41 ± 0.48 | 1.73 ± 0.12 | 1.34 ± 0.66 | 0.2614 | 0.0743 | 0.6069 |
| TARC | 0.72 ± 0.39 | 4.88 ± 1.90 | 2.60 ± 1.01 | 0.0163 (*) | 0.2433 | 0.1474 |
| TIMP-1 | 21.12 ± 18.29 | 1297.59 ± 443.64 | 476.21 ± 234.11 | 0.004 (**) | 0.2125 | 0.0307 (*) |
| TNFα | - | - | - | - | - | - |
| VEGF | 6.04 ± 1.69 | 12.49 ± 3.63 | 9.09 ± 2.62 | 0.0648 | 0.4203 | 0.3528 |
| 6Ckin/Exodus | 850.3 ± 210.8 | 955.1 ± 64.56 | 642.0 ± 101.7 | >0.9999 | 0.5391 | 0.2209 |
| TGF-β1 | 62.12 ± 60.71 | 87.47 ± 10.29 | 16.47 ± 4.25 | 0.6765 | 0.3278 | 0.0004 (***) |
| TGF-β2 | 8.89 ± 3.76 | 65.62 ± 28.09 | 26.01 ± 14.47 | 0.0214 (*) | 0.5262 | 0.0854 |
| TGF-β3 | 1.88 ± 0.43 | 19.13 ± 5.97 | 6.37 ± 3.56 | 0.0046 (**) | 0.4130 | 0.0190 (*) |
